# Toxic Effects of Two Redox States of Thallium on Immortalised Hypothalamic GT1-7 Neuronal Cells

**DOI:** 10.3390/ijms241411583

**Published:** 2023-07-18

**Authors:** Dai Mizuno, Masahiro Kawahara, Keiko Konoha-Mizuno, Terumasa Ogawara, Ryoji Hama, Kentaro Yamazaki

**Affiliations:** 1 Department of Forensic Medicine, Faculty of Medicine, Yamagata University, 2-2-2 Iida-Nishi, Yamagata-shi 990-9585, Japan; k_mizuno@med.id.yamagata-u.ac.jp (K.K.-M.); t.ogawara@med.id.yamagata-u.ac.jp (T.O.); hama-ryoji@med.id.yamagata-u.ac.jp (R.H.); yamazaki@med.id.yamagata-u.ac.jp (K.Y.); 2 Research Institute of Pharmaceutical Sciences, Faculty of Pharmacy, Musashino University, 1-1-20 Shin-machi, Nishitokyo-shi 202-8585, Japan; makawa@musashino-u.ac.jp

**Keywords:** thallium, neurotoxicity, oxidative stress, apoptosis

## Abstract

Thallium (Tl), is a highly toxic heavy metal that exists in monovalent (Tl(I)) and trivalent (Tl(III)) ionic states. This study aimed to compare the toxicities of Tl(I) and Tl(III) in a mouse hypothalamic GT1-7 neuronal cell line. Decreased viability and increased cytotoxicity were observed in the GT1-7 cells 16 h after Tl(I) or Tl(III) treatment. Tl(III) was more cytotoxic, than Tl(I), as indicated by extracellular lactate dehydrogenase levels. Both treatments induced caspase 3 activity, DNA fragmentation, malondialdehyde (MDA) production, and superoxide dismutase activity in the cells. MDA production was higher after Tl(III) than after Tl(I) treatment. Moreover, co-treatment with antioxidants, such as mannitol, ascorbic acid, or tocopherol, significantly attenuated the Tl-induced decrease in GT1-7 cell numbers. Therefore, both treatments induced oxidative stress-related apoptosis. Furthermore, Tl(III) reduced the cell viability more subtly than Tl(I) after 1 and 3 h of treatment. This effect was enhanced by co-treatment with maltol or citric acid, which promoted the influx of metallic elements into the cells. Thus, Tl(III) entered GT1-7 cells later than Tl(I) and had a delayed onset of toxicity. However, Tl(III) likely produces more extracellular lipid peroxides, which may explain its stronger cytotoxicity.

## 1. Introduction

Thallium (Tl) is a heavy metal present in trace amounts in nature. Areas with high Tl levels include Poland, Spain, Turkey, Switzerland, and China [1]. Tl toxicity is high and it is used with arsenic (As) as a poison [2]. In Japan, Tl sulphate, Tl acetate, and Tl nitrate are designated as deleterious substances under the Poisonous and Deleterious Substances Control Law. Tl can be a monovalent (Tl(I)) or trivalent ion (Tl(III)); Tl(I) is preferred in aqueous solutions, whereas Tl(III) is more stable in organic compounds and in oxidised water [3,4]. Tl(I) is used in scintillation counters for measuring radiation and rodenticides. Tl(I) salt has been used as a raw material in depilatories owing to its depilatory effect. In addition, Tl salts are still widely used as rodenticides and insecticides in certain countries, despite the World Health Organization’s 1973 recommendations to ban their use. Despite their toxicity, Tl compounds remain available in many countries worldwide [5]. Tl(III) is used in the manufacture of optical glasses, low-temperature thermometers, and semiconductors. The demand for Tl is increasing due to the widespread use of high-tech equipment. Tl is traditionally released into the environment (e.g., river water and fly ash) as a waste product of zinc (Zn), cadmium (Cd), and lead (Pb) mining and coal combustion [6]. In addition, the amount of Tl released into the environment has increased because of recent industrial demand and mining volume increases. This effluxed Tl may be taken up by plant roots and stored in the plant biomass, resulting in the entry of Tl into the food chain [7]. The increased Tl content in the human body is mainly caused by consuming Tl-contaminated food and drinking water. Tl can also enter the human body via inhalation upon exposure to airborne contaminants (fly ash) [7,8,9,10]. It is reported that concentrations of Tl in human organs follow this order: brain (0.42–1.5 ng/g) < liver (1.5 ng/g) < kidney (6.1 ng/g) < hair (150–650 ng/g) < bone (600 ng/g) < nail (1200 ng/g) [7]. However, a study in mice reported that the brain is the organ with the highest Tl(I) concentration over time [11]. Tl(I) causes injury to the human central, peripheral, and autonomic nervous systems [4,12]. A previous study compared the toxicities of Tl(III) and Tl(I) in liver cells [13]. However, their toxicities in the central nervous system (CNS) have yet to be compared.

Using a toxicity evaluation test, we previously reported that Zn and aluminium (Al) induce apoptosis in mouse hypothalamic GT1-7 neuronal cells [14,15]. These cytotoxic effects may play important roles in developing cerebrovascular dementia and Alzheimer’s disease. GT1-7 cells possess several neuronal characteristics, including neurite extension and gonadotropin hormone-releasing hormone (GnRH) secretion. They can also express receptors and neuron-specific proteins, including E2 receptors (ERα and ERβ subtypes), microtubule-associated protein 2, tau protein, neurofilament, synaptophysin, GABA_A_ receptors, dopamine receptors, and L-type Ca^2+^ channels [16]. These properties make the GT1-7 cell line an excellent model system for investigating neurotoxicity and endocrine disruption [15,17]. The hypothalamus is the center of instinctive behaviours, such as eating, sexual, and sleep, as well as emotional behaviours such as anger and anxiety. GnRH and other hormones released from the hypothalamus affect the pituitary gland and reproductive organs. Therefore, GT1-7 cell cytotoxicity can serve as an index of the effects on sleep disorders, eating disorders, and emotional behaviours. Moreover, it is reported that the highest Tl concentration in the brain was observed in the hypothalamus [4].

GT1-7 cells are useful for analysing the mechanisms of Tl toxicity in the brain and nervous system. In this study, we aimed to compare the cytotoxicities and mechanisms of action of Tl(I) and Tl(III) in GT1-7 cells. Tl(I) increases reactive oxygen species (ROS) formation and phospholipid peroxidation [18]. Therefore, we investigated the involvement of oxidative stress in GT1-7 cytotoxicities via Tl(I) and Tl(III) by co-treatment with antioxidants and evaluation of oxidative stress markers.

## 2. Results

### 2.1. Cytotoxicities of Tl(I) and Tl(III) in GT1-7 Cells

**Figure 1:**
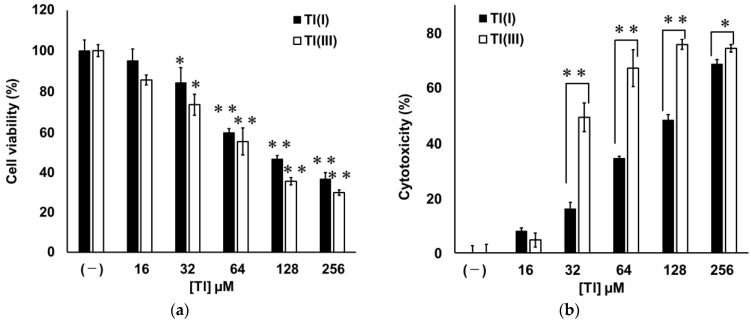
GT1-7 cytotoxicity by Tl(I) and Tl(III). GT1-7 cells were treated with various concentrations (16–256 µM) of Tl(I) (black bars) or Tl(III) (white bars), and cell viability and cytotoxicity after 16 h were measured by using the WST-1 (**a**) and lactate dehydrogenase assays (**b**). Data are expressed as mean ± standard error (*n* = 6). * *p* < 0.05, ** *p* < 0.01 vs. control (–) group (**a**) or vs. Tl(I) group (**b**).

GT1-7 cells were treated with various concentrations of Tl(I) (hereafter denoted as Tl(I) group) or Tl(III) (hereafter denoted as Tl(III) group) for 16 h. Cell viability and cytotoxicity were evaluated using WST-1 and lactate dehydrogenase (LDH) assays, respectively. Both treatments decreased the viability of GT1-7 cells in a concentration-dependent manner. The viabilities of the cells treated with Tl(I) or Tl(III) at ≥64 µM were significantly lower than that of untreated cells (*p* < 0.01). No significant difference in cell viability was observed between cells treated with Tl(I) and Tl(III) at the same concentrations (Figure 1a). In contrast, Tl(I) and Tl(III) showed dose-dependent increases in cytotoxicity. Tl(I) at ≥64 µM and Tl(III) at ≥32 µM showed significantly different cytotoxicities (*p* < 0.01) from the control (Figure 1b). Tl(III) showed significantly higher cytotoxicity than Tl(I) at concentrations of ≥32 µM.

### 2.2. Mechanisms of GT1-7 Cytotoxicity by Tl(I) and Tl(III)

**Figure 2:**
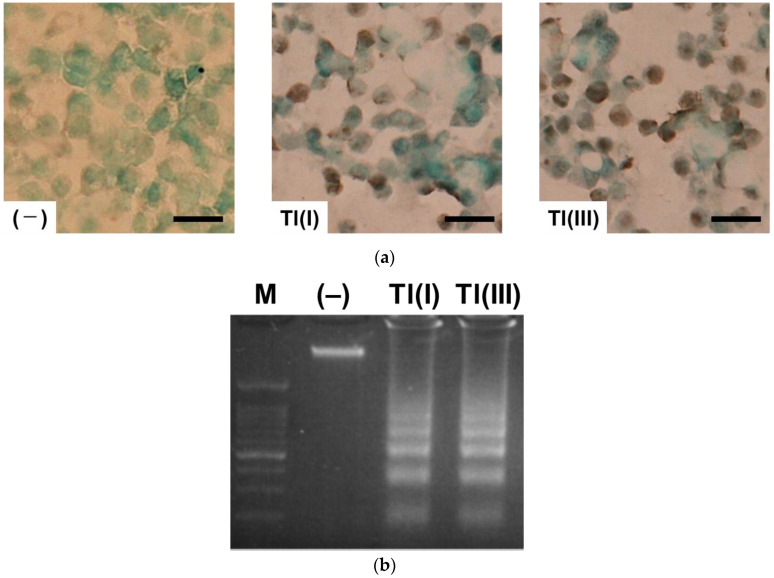
Evaluation of apoptosis in GT1-7 cells treated with Tl(I) and Tl(III) through TUNEL staining (**a**) and observation of ladder-like DNA fragmentation (**b**). GT1-7 cells were treated with 64 µM Tl(I) or Tl(III) for 16 h. (**a**) TUNEL-positive cells are visible as brown-stained nuclei. Scale bar  =  100 μm. (**b**) DNA isolated from 5 × 10^6^ cells was loaded in each lane. M, molecular weight marker; (–), control (untreated cells).

Caspase 3 activity was measured 16 h after treatment of GT1-7 cells with Tl(I) or Tl(III). Both Tl(I) and Tl(III) significantly increased caspase 3 activity (*p* < 0.01). This activity in GT1-7 cells treated with 64 µM Tl(I) or Tl(III) was 4.0- and 3.6-fold higher than that in the control group, respectively (Table 1). No significant differences in caspase 3 activity were observed between the Tl(I) and Tl(III) groups. In addition, terminal deoxynucleotidyl transferase-mediated dUTP nick-end labelling (TUNEL)-positive cells were detected in Tl(I) and Tl(III) groups (Figure 2a), and the DNA prepared from these cells was fragmented in a ladder-like pattern (Figure 2b). These results suggest that Tl(I) and Tl(III) induce apoptosis in GT1-7 cells.

### 2.3. Tl(I) and Tl(III) Cause Oxidative Stress in GT1-7 Cells

We investigated whether oxidative stress, a representative stress that induces apoptosis, is involved in Tl-induced GT1-7 cell death. Superoxide dismutase (SOD) activity, intracellular and extracellular reduced/oxidised glutathione (GSH/GSSG), which are markers of oxidative stress, and malondialdehyde (MDA) production, which is an index of lipid peroxidation, were measured 16 h after treatment with Tl(I) or Tl(III). SOD activity increased by 3.4- or 8.2-fold, and MDA production increased by 2.9- or 4.3-fold in the Tl(I) or Tl(III) group, respectively, compared to the control group (Table 1, 16 h). Meanwhile, Tl(I) and Tl(III) significantly increased GSSG levels (*p* < 0.05: Tl(I), *p* < 0.01: Tl(III)) and significantly decreased GSH levels (*p* < 0.05: intracellular Tl(I), *p* < 0.01: extracellular Tl(I); intracellular and extracellular Tl(III)), both intracellularly and extracellularly (Table 2, 16 h). Treatment with Tl(III) induced significantly higher SOD activity and MDA production than did treatment with Tl(I). Extracellularly, GSSG levels were higher, whereas GSH levels were lower in the Tl(III) group than in the Tl(I) group. In addition to these results, ROS production was measured 16 h after treatment with Tl(I) or Tl(III). ROS production significantly increased by 3.7- or 3.9-fold in the Tl(I) or Tl(III) group (16 h in Figure S1). These results suggest that treatment with Tl(I) or Tl(III) induces oxidative stress in GT1-7 cells and that the oxidative stress induced by Tl(III) is stronger than that induced by Tl(I).

**Figure 3:**
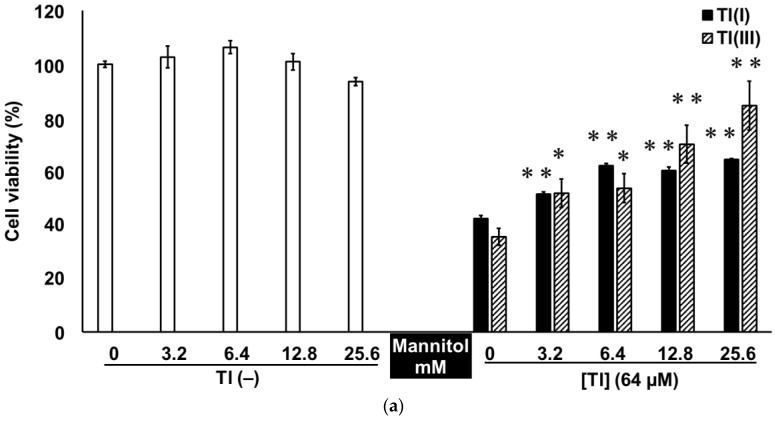

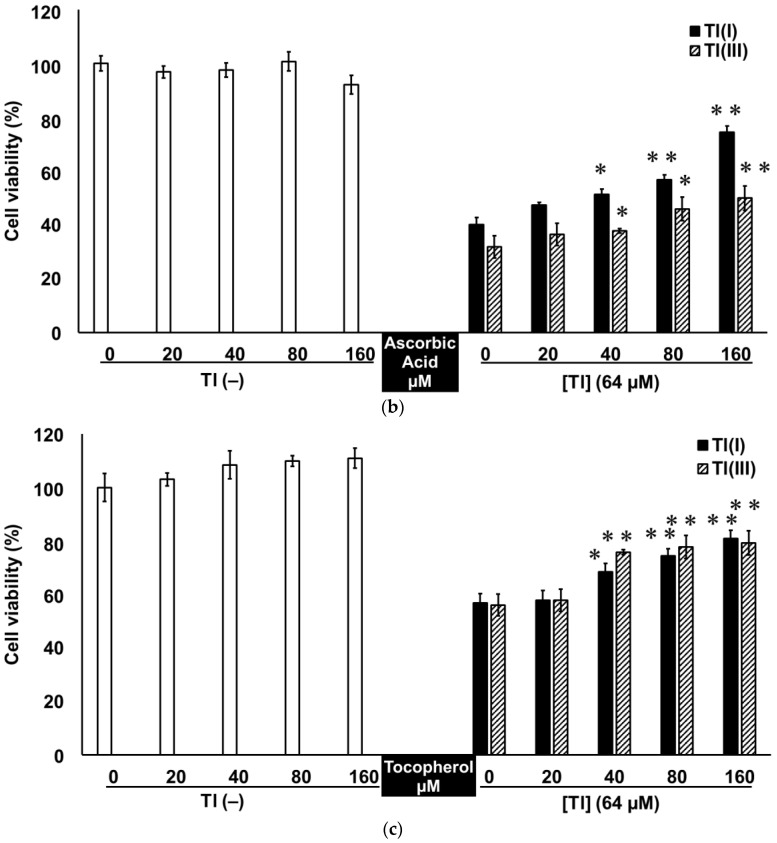
Effect of antioxidants on Tl-induced cytotoxicity. Mannitol (**a**), ascorbic acid (**b**), or tocopherol (**c**) was added to GT1-7 cells in the presence or absence (white graph) of 64 µM Tl(I) (black graph) or Tl(III) (shaded graph). Cell viability was analysed 16 h later using the WST-1 assay. Data represent the mean ± standard error of the mean (*n* = 6). * *p* < 0.05, ** *p* < 0.01 vs. the non-antioxidant group.

The antioxidant D-mannitol (mannitol), DL-α-tocopherol (tocopherol), or L-ascorbic acid (ascorbic acid) was co-treated with Tl(I) or Tl(III) to confirm the effect of oxidative stress on Tl-induced GT1-7 cytotoxicity. The viability, caspase 3 activity, MDA production, and SOD activity in GT1-7 cells were evaluated 16 h after co-treatment. In the absence of Tl, these antioxidants did not significantly alter the viability of GT1-7 cells (Figure 3). Co-treatment with mannitol (Figure 3a), ascorbic acid (Figure 3b), or tocopherol (Figure 3c) with 64 µM Tl(I) or Tl(III) significantly increased the survival rate of the GT1-7 cells (Figure 3a). Moreover, all of these antioxidants significantly inhibited caspase 3 activity (Table 1) induced by treatment with Tl(I) or Tl(III). Suppression of MDA production and SOD activity was also observed in the cells co-treated with antioxidants and Tl (Table 2). The reduction in oxidative stress by these antioxidants may have contributed to the alleviation of GT1-7 cytotoxicity by Tl(I) and Tl(III). These results suggest that Tl(I) and Tl(III) induce apoptosis associated with oxidative stress in GT1-7 cells.

### 2.4. Time Course Change of GT1-7 Cytotoxicity by Tl(I) and Tl(III)

**Figure 4:**
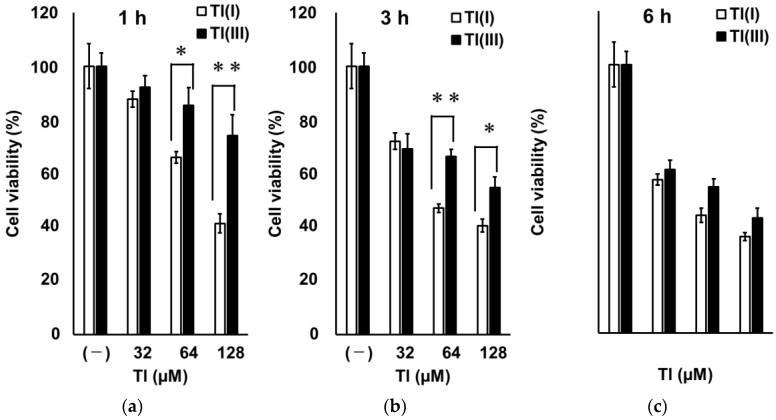
Time course of GT1-7 cell viability after treatment with Tl(I) or Tl(III). GT1-7 cells were treated with various concentrations (32–128 µM) of Tl(I) (white graph) or Tl(III) (black graph). After 1 (**a**), 3 (**b**) and 6 h (**c**), cell viability and cytotoxicity were analysed using the WST-1 assay. Data represent the mean ± standard error of the mean (*n* = 6). * *p* < 0.05, ** *p* < 0.01.

**Figure 5:**
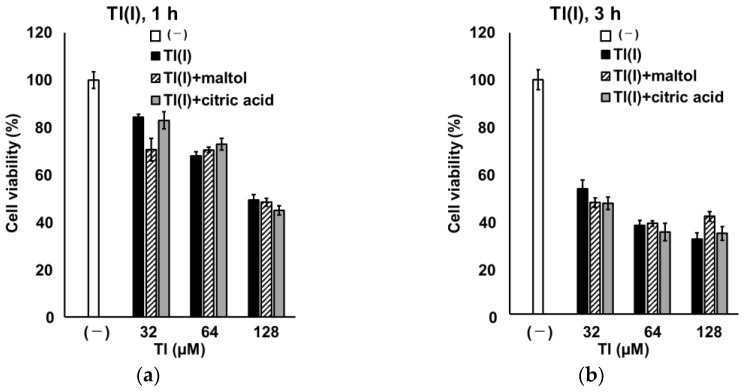

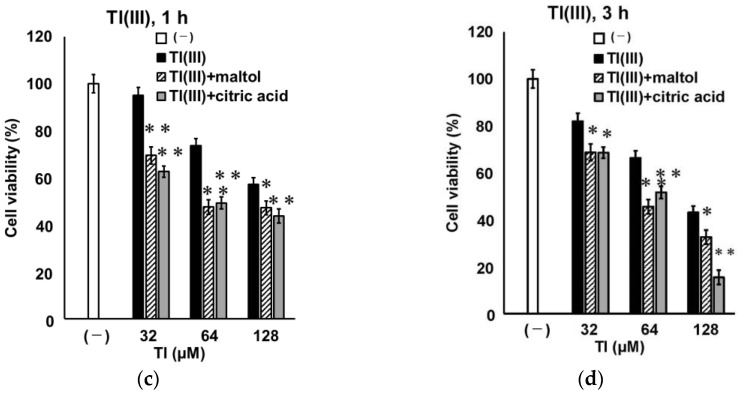
Effects of citric acid and maltol on Tl-induced cytotoxicity. Tl(I) (**a**,**b**) or Tl(III) (**c**,**d**) was mixed with maltol (hatched line graph) or citric acid (gray graph) and used to treat GT1-7 cells at each concentration shown in the figure (32–128 µM). At 1 (**a**,**c**) or 3 (**b**,**d**) h after treatment, cell viability was analysed using the WST-1 assay. Data are expressed as mean ± standard error (*n* = 6). * *p* < 0.05, ** *p* < 0.01 vs. Tl only group (black graph).

To compare the rapidity of GT1-7 cytotoxicity induced by Tl(I) and Tl(III), we investigated GT1-7 cytotoxicity at earlier time points (1, 3, and 6 h) after treatment. Considering that LDH activity could not be measured after 1, 3, and 6 h under the conditions used in this study, we evaluated GT1-7 cytotoxicity by comparing cell viability using WST-1 assay. The viability of Tl(I)- and Tl(III)-treated GT1-7 cells decreased in a dose-dependent manner at each examined time point (Figure 4). At 1 and 3 h after treatment, cell viability was significantly lower in the Tl(I) group than in the Tl(III) group (Figure 4a,b). At 6 h after treatment, Tl(I) and Tl(III) groups showed similar decreases in cell viability (Figure 4c). We also investigated the increase in intracellular and extracellular GSSG, extracellular GSH consumption (Table 2; 1 and 3 h), and MDA production (Table 3). These parameters were significantly higher in Tl(III) group than in Tl(I) group. Additionally, the ROS level peaked at 1 h after treatment in the Tl(I) group and at 3 h in the Tl(III) group (Figure S1). The peak of ROS level in the Tl(III) group (10.0-fold vs. untreated group) was significantly higher (*p* < 0.01) than that of the Tl(I) group (4.9-fold untreated group). The increase in ROS levels decreased after 6 h, possibly due to Tl-induced cell decrease. Furthermore, a mixture of Tl(I) or Tl(III) with citric acid or maltol, which promotes the influx of metals, such as Al, into cells, was administered to GT1-7 cells. Cell viability and MDA production were assessed after 1 or 3 h of treatment. Neither citric acid nor maltol significantly affected the decrease in GT1-7 cell viability or MDA production following Tl(I) treatment (Figure 5a,b). However, in the presence of Tl(III), these chelators significantly enhanced the decrease in GT1-7 cell viability (Figure 5c,d) and significantly inhibited MDA production (Table 3). GT1-7 cytotoxicity by Tl(III) occurred later than that by Tl(I), which may be due to the slower entry rate of Tl(III) into the cells than Tl(I).

## 3. Discussion

Tl is a highly toxic metal that damages various organs, including the CNS [6,8,9,10,12,13]. The demand for this metal, especially Tl(III), is increasing in the high-tech and future industrial technology fields because it is used in optical glass and high-temperature superconductors. Tl-related poisoning cases will increase in the future, which is a concern currently. To investigate the possible toxicity of Tl(I) and Tl(III) to the CNS, we compared the toxicities of these two ion states in the neuronal model GT1-7 cells. Treatment with Tl(I) or Tl(III) decreased the viability of GT1-7 cells in a dose-dependent manner. The LDH assay results also showed that the cytotoxicities of Tl(I) and Tl(III) increased in a concentration-dependent manner. These results suggested that the decrease in cell viability was due to the cytotoxicity of Tl(I) or Tl(III) rather than the suppression of cell proliferation. Cytotoxicity of Tl(I) or Tl(III) was observed at conditions close to the minimum lethal dose [4] of Tl(I) in humans (15 mg/kg; approximately 30 µM) (Figure 1). Induction of caspase 3 activity, ladder-like DNA fragmentation, and TUNEL-positive cells were observed in the Tl(I) and Tl(III) groups, suggesting that both ionic states of Tl-induced apoptosis in GT1-7 cells. At this time, increases in SOD activity, MDA production, intracellular and extracellular GSSG levels, and extracellular GSH consumption were observed. Moreover, GT1-7 cytotoxicity by Tl(I) and Tl(III) was ameliorated by co-treatment with antioxidants, such as mannitol, ascorbic acid, and tocopherol. Under the conditions used in this study, the antioxidants were not cytotoxic (Figure 3). Individual oral administration of 1250 mg of ascorbic acid, 1000 mg of tocopherol, and 150 g of mannitol produced peak plasma concentrations of 134.8 µM, 6.78 µM, and 7.09 mM, respectively [19,20,21]. These antioxidants are non-toxic, particularly ascorbic acid, which attenuates the cytotoxicity of Tl at physiologically relevant concentrations. These results indicate that both Tl states induce oxidative stress in GT1-7 cells. Oxidative stress causes apoptosis [22,23,24,25,26] and increases the production of ROS, which plays an important role in Tl-induced toxicity. Tl increases oxidative stress by inducing lipid peroxidation in tissues, such as the brain, liver, and thymus, and this process is an important risk factor for tissue damage and organ dysfunction [13,27,28]. In this study, these phenomena could be the cytotoxic mechanisms of Tl in GT1-7 cells. The cells are thus a useful model for analyzing the toxic mechanisms of Tl in the brain and nervous system.

Treatment with Tl(I) at 64 and 128 µM significantly decreased the viability of GT1-7 cells after 1 h (*p* < 0.01 vs. (–) the group in Figure 4a). The decrease in GT1-7 cell viability induced by Tl(III) treatment was significantly lower than that induced by Tl(I) at 1 and 3 h (Figure 4a,b), suggesting that Tl(III)-induced cytotoxicity occurred later than Tl(I)-induced cytotoxicity. The chemical properties of Tl(I) and Tl(III) are similar to those of potassium (K) and Al, respectively [29]. Tl(I) behaves similarly to K in vivo and competes with K in cell membranes and mitochondria in K-rich organs, such as nerves, liver, and myocardium, at a concentration 1/10 that of K; it also exerts toxic effects by interfering with K-dependent cellular processes [3]. A previous study speculated that Tl(I), similar to K, rapidly influxes into cells due to the actions of sodium-potassium pumps [29], and thus exhibits GT1-7 cytotoxicity relatively early. However, the bioavailability of Al, which is similar to that of Tl(III), is generally much lower than that of K [30]. Al associates with citric acid citrate or forms a chelate with maltol, promoting its accumulation in the brain and uptake into cultured neuronal cells [31,32,33]. Al citrate is the major species present in the extracellular fluid of the brain (approximately 60%) [34]. It was reported that the presence of a transporter promotes the uptake of Al citrate into cells in a Na^+^- and ATP-independent manner [33]. Maltol forms hydrolytically stable and hydrophilic complexes with Al^3+^ [35]. This highly toxic complex enhances Al accumulation in cultured cells [36]. These compounds can enhance the cellular uptake and toxicity of Tl, which has properties similar to those of Al. Co-treatment of citric acid or maltol with Tl(III) further decreased cell viability at 1 and 3 h (Figure 5c,d), suggesting that Tl(III) enters GT1-7 cells like that of Al. Such differences in the behaviour of Tl(I) and Tl(III) affect the influx rates into GT1-7 cells under these ionic states, which may explain the different rates of toxicity.

This study showed that both Tl statuses could induce oxidative stress in GT1-7 cells. SOD activity, MDA production, GSSG increase, and GSH depletion at 1, 3, and 16 h after treatment were significantly higher in the Tl(III) group than in those the Tl(I) group (Table 1, Table 2 and Table 3). In addition, the increase in MDA production due to Tl(III) treatment was significantly reduced by co-treatment of Tl(III) with citric acid or maltol (Table 3). Although factors that include SOD protect cells against internal oxidative stress, few protective factors exist outside cells, making cells vulnerable to external oxidative stress. Tl(III) enters GT1-7 cells more slowly than Tl(I). Thus, it prolongs the exposure of membrane lipids to oxidative stress and increases lipid peroxide production. This stronger extracellular oxidative stress due to Tl(III) treatment may explain the stronger GT1-7 cytotoxicity of Tl(III) than that of Tl(I) (Figure 1b).

K and Al cross the blood-brain barrier in mammals. Tl(I) and Tl(III), which have properties similar to these metals, can cause severe acute and chronic damage to the CNS [12]. In particular, Al accumulates in the hippocampus and is believed to cause cognitive impairment, dementia, and accelerated brain aging [37,38,39]. Exposure to Tl(III) can damage the CNS through mechanisms similar to those for Al. Epidemiological studies have reported a positive correlation between environmental Al concentrations and cognitive impairment [40]. We reported that Al overdose cytotoxicity in GT1-7 cells might be involved in the development of neurological diseases, such as Alzheimer’s disease [14,31]. Comparing the mechanism of GT1-7 cytotoxicities induced by Tl(III) with that induced by Al may provide a powerful clue for elucidating cranial neuropathy induced by Tl(III). Further investigation of the toxic mechanisms induced by Tl, especially Tl(III), in GT1-7 cells is warranted to elucidate the mechanisms of action of Tl in the CNS.

## 4. Materials and Methods

### 4.1. Reagents

**Figure 6:**
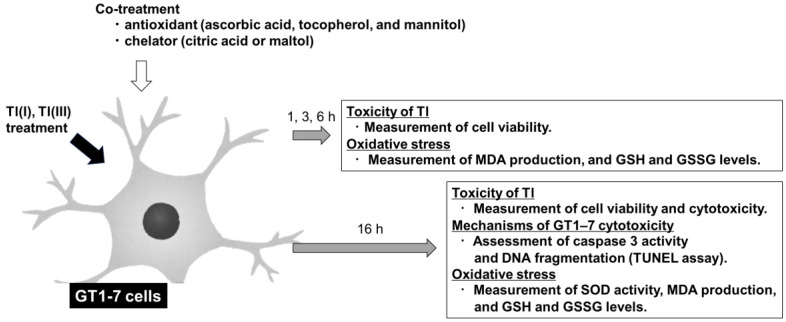
Schematic diagram of the experiments. GT1-7 cells were treated with Tl(I) and Tl(III) in the presence or absence of an antioxidant or chelator. Cell viability and oxidative stress markers (MDA, GSH, and GSSG levels) were evaluated at 1, 3, 6, and 16 h after Tl treatment. At 16 h after Tl treatment, cell viability, cytotoxicity, caspase-3 activity, DNA fragmentation, and oxidative stress markers (SOD, MDA, GSH, and GSSG levels) were evaluated.

Dulbecco’s Modified Eagle’s medium/F-12HAM (DMEM/F-12), DMEM/F-12 HEPES buffer (DMEM/F-12/HEPES) were purchased from Sigma-Aldrich (St. Louis, MO, USA). Tocopherol and ascorbic acid were purchased from Tokyo Kasei Kogyo Co., Ltd. (Tokyo, Japan). Thallium(III) nitrate and D-mannitol were purchased from Yoneyama Yakuhin Kogyo (Osaka, Japan). Thallium(I) sulphate and other reagents used in the experiments were purchased from Fujifilm Wako Pure Chemical Co., Ltd. (Osaka, Japan). Tl(III) was prepared as a 3M HCl solution immediately before use and diluted with pure water to the concentration used and the hydrochloric acid concentration being 1 mM or less. Tocopherol was dissolved in ethanol, and the other reagents were dissolved in Milli-Q water. A schematic of the experimental flow is shown in Figure 6.

### 4.2. Cell Culture

GT1-7 cells (provided by Dr. R. Weiner, University of California at San Francisco, San Francisco, CA, USA) were grown in DMEM/F-12 supplemented with 10% foetal bovine serum. After enzymatic digestion with trypsin, cells were resuspended in serum-free DMEM/F-12/HEPES and plated onto culture plates [41]. Cells were then cultured in a humidified incubator at 37 °C and 5% CO_2_. Cells used for the WST-1 and LDH assays were cultured at 5 × 10^4^ cells per well (200 μL) in a 96-well plate. In addition, cells were cultured in a 10 cm cell culture dish at 5 × 10^6^ cells and used for caspase 3 activity measurement, DNA ladder detection, SOD activity measurement, MDA measurement, or GSH/GSSG measurement.

### 4.3. Evaluation of Cytotoxicity

Cytotoxicity was evaluated using WST-1 and LDH assays. GT1-7 cells were cultured in a 96-well plate for 24 h under serum-free conditions, and 1 μL of Tl(I) or Tl(III) was added as a solution and adjusted to the final concentration shown in each figure. Milli-Q water (1 μL) was used as the Tl(I) control, and 1 μL of 1 mM HCl was used as the Tl(III) control. The cell viability and LDH activity in the group treated with 1 mM HCl were not significantly different from those in the group treated with Milli-Q water. At 1, 3, 6, and 16 h after Tl treatment, 100 µL of the supernatant was transferred to a new 96-well plate, and cytotoxicity was evaluated by an LDH assay using the cytotoxicity LDH assay kit WST (Dojindo Laboratories, Kumamoto, Japan) according to the manufacturer’s instructions. To calculate cell viability, the remaining cells in the medium were counted using the WST-1 assay with a cell counting kit-8 (Dojindo Laboratories) following the manufacturer’s instructions. Mannitol, ascorbic acid, and tocopherol were added with 64 µM Tl(I) or Tl(III) at each concentration (Figure 3), and cell viability was evaluated after 16 h. Citric acid or maltol was mixed with Tl(I) or Tl(III) at a molar ratio of 1:1 or 1:3, respectively, and cell viability was evaluated after 1 and 3 h. The mixing ratio of citric acid and maltol to Tl was determined based on previous reports using Al [32,33,34]. The absorbance of the treated samples and the fluorescence of control and treated cells was against a blank control using an iMark™ microplate absorbance reader (Bio-Rad Laboratories, Hercules, CA, USA). The WST-1 and LDH assays were performed at wavelengths of 450 and 490 nm, respectively. In the WST-1 assay, cell viability was determined by dividing the absorbance of each treated group at 450 nm by that of the control group. In the LDH assay, untreated cells were used as the low control group, and cells cultured for 30 min after treatment with the lysis buffer attached to the kit were used as the high control group. When the cell viability of the high control group was evaluated using WST-1, it was almost 0%. Cytotoxicity (%) was calculated using the following formula: cytotoxicity (%) = [(each treatment group) − (low control group)]/[(high control group) − (low control group)].

### 4.4. Determination of Caspase 3 Activity

Caspase 3 activity in the Tl group was measured using a caspase 3 colorimetric assay kit (BioVision Inc., Milpitas, CA, USA). Dissociated cells were plated onto 10 cm culture dishes at a concentration of 5 × 10^6^ cells/dish in a 10 mL culture medium. After 24 h of incubation, the cells were treated with semi-lethal concentrations of Tl(I) or Tl(III) (64 µM). The Tl(I) control was treated with 50 µL of pure water, and Tl(III) control was treated with 50 µL of 1 mM HCl. Additionally, 25.6 mM mannitol, 160 µM ascorbic acid, or 160 µM tocopherol was treated with Tl(I) or Tl(III). At 16 h after treatment, the cells were collected using a cell scraper, suspended in a cell lysis buffer attached to the kit, and allowed to stand on ice for 10 min. The cells were centrifuged at 10,000× *g* for 1 min at 25 °C, and the supernatant was used as the measurement sample. Caspase 3 activity was measured according to the manufacturer’s instructions. The absorbance was measured at 405 nm using the aforementioned iMark reader. Caspase 3 activity was determined by dividing the absorbance of the Tl solution-treated group at 405 nm by that of the control group.

### 4.5. TUNEL Staining

TUNEL staining was employed to demonstrate apoptotic cells. GT1-7 cells treated with Tl(I) and Tl(III), similar to the measurement of caspase 3 activity, were harvested with a cell scraper after 16 h. After washing twice with PBS, the cells were suspended in PBS containing 10% formalin. Cells (1 × 10^5^) were plated on poly-D-lysine-coated glass slides. TUNEL assays were performed using apoptosis in situ detection kit Wako (Fujifilm Wako Pure Chemical Co., Ltd.) following the manufacturer’s instructions. As a negative control, cells were incubated with the labelling reaction solution without the TdT enzyme, resulting in no TUNEL positivity. Cell nuclei were counterstained with a 0.5% (*v*/*v*) methyl green solution for 5 min at approximately 25 °C.

### 4.6. DNA Fragmentation Assay

A DNA fragmentation assay was used to assess the abilities of Tl(I) and Tl(III) to induce genomic DNA damage in GT1-7 cells. Dissociated cells were plated onto 10 cm culture dishes at a concentration of 5 × 10^6^ cells/dish in a 10 mL of culture medium. After 24 h of incubation, the cells were treated with semi-lethal concentrations of Tl(I) or Tl(III) (64 µM). After 16 h of exposure, DNA extraction and purification were performed using the ApopLadder Ex™ kit from TaKaRa Bio Inc. (Shiga, Japan) following the manufacturer’s instructions. The eluent containing the DNA pellets was subjected to electrophoresis on a 2.0% agarose gel at 100 V for 90 min. DNA bands were visualised and photographed using an ultraviolet gel documentation system.

### 4.7. Measurement of SOD Activity

SOD activity in the Tl group was measured using the SOD assay kit WST (Dojindo Laboratories) following the manufacturer’s instructions. GT1-7 cells that were treated similarly to those used to measure caspase 3 activity were harvested with a cell scraper after 16 h. After washing twice with PBS, the cell membrane was disrupted using a homogeniser, and 1 mL of PBS was added and mixed. The cells were centrifuged 10,000× *g* for 15 min at 4 °C, and the supernatant was used as the measurement sample. Each sample was serially diluted twofold with the dilution buffer attached to the kit and added to a 96-well plate. Absorbance was measured at 450 nm using the aforementioned iMark reader, and SOD activity was calculated using the formula described in the manufacturer’s instructions.

### 4.8. Measurement of MDA Production

The amount of MDA produced was evaluated using the OxiSelect™ TBARS assay kit (MDA Quantitation) (Cell Biolabs, Inc., San Diego, CA, USA). Tl(I) or Tl(III) at 64 µM treated GT1-7 cells were cultured similarly for measuring caspase 3 activity. Cells were collected using a cell scraper at 1, 3, and 16 h after treatment. In addition, 25.6 mM mannitol, 160 µM ascorbic acid, or 160 µM tocopherol were co-treated with Tl(I) or Tl(III), and the cells were harvested using a cell scraper 16 h after treatment. Furthermore, citric acid or maltol was mixed with Tl(I) and Tl(III) at molar ratios of 1:1 or 1:3, respectively, and used to treat GT1-7 cells. Cells were collected using a cell scraper at 1 or 3 h after treatment. After washing twice with PBS, the cells were resuspended in PBS at a density of 1 × 10^7^ cells/mL, and measured according to the manufacturer’s instructions. The absorbance was measured at 532 nm using the aforementioned iMark reader.

### 4.9. Measurement of GSH and GSSG Levels

GSH and GSSG levels were determined using a GSSG/GSH Quantification Kit (Dojindo Laboratories). Tl(I) or Tl(III) at 64 µM was used to treat GT1-7 cells cultured similarly for measuring caspase 3 activity. Cells and medium were harvested with a cell scraper at 1, 3, and 16 h after treatment. The cells were separated from the supernatant by centrifugation at 200× *g* for 10 min, and the supernatant was used as the extracellular sample. The cells were washed twice with PBS, dispersed in 80 µL of 10 mM HCl, and freeze-thawed twice to disrupt the cell membranes. Next, the cells were added to 20 µL of 5% 5-sulfosalicylic acid and then centrifuged at 8000× *g* for 10 min. The supernatant was used as an intracellular sample. The GSH and GSSG levels were measured according to the manufacturer’s instructions. The absorbance was measured at 405 nm using the aforementioned iMark reader.

### 4.10. Statistical Analyses

All experimental results were tested for significance using Dunnett’s multiple comparison method with the EZR statistical software version 1.61 (Saitama Medical Center, Jichi Medical University, Saitama, Japan) [42]. Statistical significance was set at *p* < 0.05.

## 5. Conclusions

The two ionic states of Tl, Tl(I) and Tl(III), can induce oxidative stress and apoptosis in GT1-7 cells. Tl(I) and Tl(III) affected GT1-7 cells like those of K and Al, respectively. In the future, the use of high-tech equipment will increase the demand for Tl(III), which is used in semiconductor manufacturing. The release of Tl into the environment is expected to increase because of an increase in the amount of mined Tl. Thus, understanding the mechanism of its toxicity is crucial because Tl poses a risk of serious CNS damage.

## Figures and Tables

**Table 1:** Levels of caspase 3 activity, superoxide dismutase (SOD) and malondialdehyde (MDA) 16 h after Tl treatment.

16 h	Caspase 3 Activity Ratio (vs. (–) Group)	SOD (Unit/mg Protein)	MDA (µM)
(–)	–	5.5 ± 0.7	4.2 ± 0.3
Tl(I) 64 µM	4.0 ± 0.0 **	18.6 ± 2.3 **	17.5 ± 1.3 **
Tl(I) 64 µM+ Ascorbic acid 160 µM	1.5 ± 0.0 ^##^	8.1 ± 1.3 ^##^	5.8 ± 0.4 ^##^
Tl(I) 64 µM + Tocopherol 160 µM	1.6 ± 0.3 ^##^	8.9 ± 2.3 ^##^	5.2 ± 0.1 ^##^
Tl(I) 64 µM + Mannitol 25.6 mM	1.9 ± 0.1 ^##^	8.1 ± 3.5 ^##^	5.6 ± 0.1 ^##^
Tl(III) 64 µM	3.6 ± 0.4 **	45.1 ± 6.2 **^,##^	26.1 ± 1.1 **^,##^
Tl(III) 64 µM + Ascorbic acid 160 µM	1.6 ± 0.0 ^$$^	10.0 ± 1.2 ^$$^	5.5 ± 0.2 ^##^
Tl(III) 64 µM + Tocopherol 160 µM	1.6 ± 0.2 ^$$^	10.4 ± 1.3 ^$$^	5.2 ± 0.1 ^##^
Tl(III) 64 µM + Mannitol 25.6 mM	1.7 ± 0.2 ^$$^	9.6 ± 1.6 ^$$^	6.0 ± 0.2 ^##^

Data represent the mean ± standard error of the mean (*n* = 6). Compared to the untreated group (–), ** *p* < 0.01. Compared to the Tl(I) group, ^##^ *p* < 0.01. Compared to the Tl(III) group, ^$$^ *p* < 0.01.

**Table 2:** Intracellular and extracellular levels of glutathione 1, 3, and 16 h after Tl treatment.

		Intracellular		Extracellular	
		GSSG	GSH	GSSG	GSH
	(–)	2.19 ± 0.24	52.57 ± 0.54	0.76 ± 0.03	2.57 ± 0.11
1 h	Tl(I) 64 µM	3.22 ± 0.15 *	51.88 ± 0.24	0.92 ± 0.06 *	2.13 ± 0.12 *
	Tl(III) 64 µM	3.85 ± 0.20 **^,#^	52.71 ± 0.50	1.33 ± 0.05 **^,##^	1.15 ± 0.12 **^,##^
	(–)	1.69 ± 0.18	54.18 ± 2.03	0.70 ± 0.04	2.75 ± 0.13
3 h	Tl(I) 64 µM	2.88 ± 0.14 **	51.51 ± 0.53 *	1.11 ± 0.04 **	2.36 ± 0.07 *
	Tl(III) 64 µM	4.60 ± 0.62 **^,#^	47.14 ± 1.81 *	1.45 ± 0.03 **^,#^	1.99 ± 0.14 **^,##^
	(–)	1.16 ± 0.05	67.52 ± 0.70	0.72 ± 0.03	11.19 ± 0.40
16 h	Tl(I) 64 µM	1.45 ± 0.08 *	63.77 ± 0.37 *	1.04 ± 0.07 *	4.44 ± 0.14 **
	Tl(III) 64 µM	1.82 ± 0.35 **	63.26 ± 0.81 **	2.04 ± 0.22 **^,##^	3.78 ± 0.31 **^,##^

Data represent the mean ± standard error of the mean (*n* = 6). Compared to the untreated group (–), * *p* < 0.05, ** *p* < 0.01. Compared to the Tl(I) group, ^#^ *p* < 0.05, ^##^ *p* < 0.01.

**Table 3:** MDA production at 1 and 3 h after Tl treatment.

MDA	1 h (µM)	3 h (µM)
(–)	3.9 ± 0.4	4.4 ± 1.3
Tl(I) 64 µM	14.0 ± 0.3 **	9.4 ± 1.1 **
Tl(I) 64 µM + Maltol 192 µM	12.6 ± 0.1 **	11.4 ± 1.2 **
Tl(I) 64 µM + Citric acid 64 µM	12.6 ± 0.2 **	10.4 ± 0.5 **
Tl(III) 64 µM	18.2 ± 0.7 **^,#^	17.5 ± 0.7 **^,#^
Tl(III) 64 µM + Maltol 192 µM	8.9 ± 0.2 **^,$$^	11.5 ± 0.6 **^,$$^
Tl(III) 64 µM + Citric acid 64 µM	11.9 ± 0.6 **^,$$^	11.5 ± 0.9 **^,$$^

Data represent the mean ± standard error of the mean (*n* = 6). Compared to the untreated group (–), ** *p* < 0.01. Compared to the Tl(I) group (–), ^#^ *p* < 0.05. Compared to the Tl(III) group (–), ^$$^ *p* < 0.01.

## Data Availability

Data supporting the findings of this study are available from the corresponding author, Dai Mizuno, upon reasonable request.

## Supplementary Materials

The following supporting information can be downloaded at: https://www.mdpi.com/article/10.3390/ijms241411583/s1.
