# Supplementary material for: Toxic Effects of Two Redox States of Thallium on Immortalised Hypothalamic GT1-7 Neuronal Cells
Source: Int J Mol Sci. 2023 Jul 18;24(14):11583. doi: 10.3390/ijms241411583 (PMC10380634; doi:10.3390/ijms241411583)
Supplement: Supplementary file 1 [file ijms-24-11583-s001.zip › ijms-2481437-supplementary.pdf]

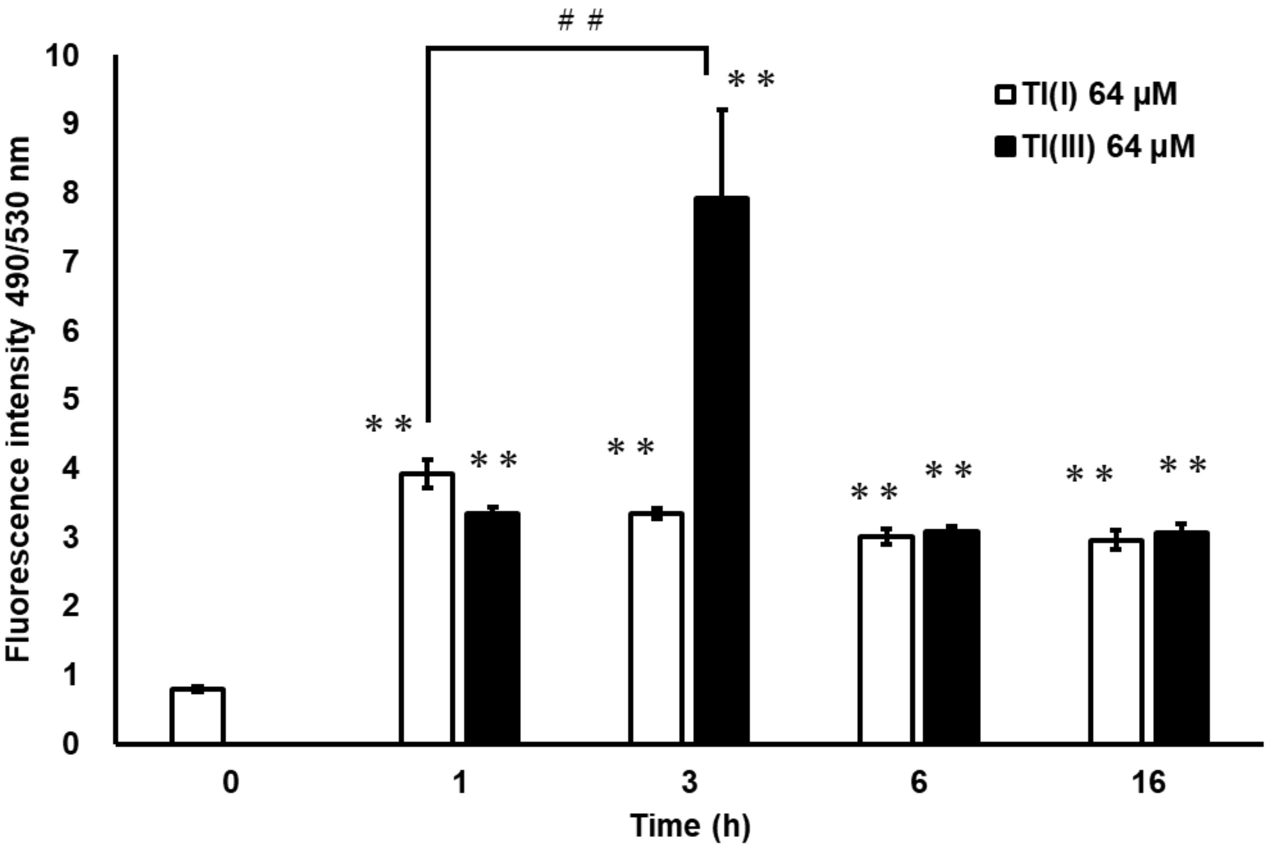

Figure S1. Levels of ROS production 1, 3, and 16 h after Tl treatment. GT1-7 cells were treated with 64 μM of Tl(I) (white graph) or Tl(III) (black graph), and ROS production after 1, 3, and 16 h were measured by using a ROS Assay Kit -Highly Sensitive DCFH-DA-. Data are expressed as mean ± standard error (n = 6). Compared to the untreated group (0 h), ** p <0.01. Compared to the Tl(I) group, ##p <0.01.

Supplementary Method

Total reactive oxygen species (ROS) measurement

Intracellular reactive oxygen species (ROS) was measured using a ROS Assay Kit -Highly Sensitive DCFH-DA- (Dojindo Laboratories). Following treatment with Tl(I) and Tl(III), similar to the evaluation of cell viability, ROS production in GT1-7 cells was measured according to the manufacturer's instructions. The fluorescence of control and treated cells was measured using a Varioskan™ Flash multimode microplate reader (Thermo Fisher Scientific Inc., Waltham, MA, USA): excitation 490 nm, emission 530 nm.
